# Effect of flywheel resistance training on change of direction performance in team sports: A systematic review and meta-analysis

**DOI:** 10.1097/MD.0000000000048524

**Published:** 2026-05-01

**Authors:** Junxin Zhang, Jing Mi, Ruidong Liu

**Affiliations:** a Sports Coaching College, Beijing Sport University, Beijing, China; b Key Laboratory of Sport Training of General Administration of Sport of China, Beijing Sport University, Beijing, China.

**Keywords:** athletic performance, biomechanical phenomena, exercise therapy, muscle strength, resistance training

## Abstract

**Background:**

Change of direction (CoD) ability is a key determinant of performance in team sports. Flywheel resistance training (FRT) has been proposed as an effective modality to improve CoD performance due to its emphasis on eccentric overload. This system review evaluates the effectiveness of FRT in enhancing CoD performance among team sports athletes.

**Methods:**

We followed PRISMA 2020. SPORTDiscus, PubMed/MEDLINE, Web of Science, and Scopus were searched through January 1, 2024 (Google Scholar screened for additional records). Eligible studies were randomized controlled or randomized crossover trials in team sport athletes, comparing FRT with non-flywheel comparators, with ≥4 weeks duration and at least 1 CoD outcome. Standardized mean differences (Hedges *g*) with 95% CIs were pooled using fixed- or random-effects models according to heterogeneity (*I*^2^). Egger’s test for small-study effects was performed when *k* ≥ 10. Study quality was appraised with the National Institutes of Health tool.

**Results:**

Meta-analysis revealed a significant enhancement in CoD performance following FRT compared to control interventions (effect size [ES] = −0.62). Notably, FRT exhibited greater efficacy among adult athletes (ES = −1.07; 95% CI = −1.51, −0.64) in contrast to those aged under 16 (ES = 0.16; 95% CI = −0.56, 0.89). Additionally, training frequencies of <2 sessions per week demonstrated a more substantial effect (ES = −1.18; 95% CI = −1.71, −0.64) compared to more frequent sessions (ES = −0.37; 95% CI = −0.93, 0.18). Moreover, extended training durations exceeding 8 weeks yielded superior outcomes (ES = −1.46; 95% CI = −2.04, −0.88) compared to shorter durations (ES = −0.44; 95% CI = −0.93, 0.04). Evaluation tests such as the *T*-test (ES = −2.10; 95% CI = −3.43, −0.77), Illinois agility test (ES = −1.19; 95% CI = −1.80, −0.57), and V-cut test (ES = −0.65; 95% CI = −0.96, −0.33) exhibited superior sensitivity in detecting CoD improvements over the shuttle run test (ES = −0.27; 95% CI = −0.82, 0.29).

**Conclusion:**

FRT is an effective strategy for improving CoD performance in team sport athletes, particularly when implemented with lower frequency and longer duration. The observed benefits are likely attributed to enhanced eccentric strength, neuromuscular efficiency, and braking capacity.

**Key Points**

1. FRT is advantageous for improving CoD performance.
2. FRT programs with frequencies <2 sessions/wk and durations exceeding 8 weeks render the most substantial COD improvement for adult team sports athletes.

## 1. Introduction

Team sports such as soccer, rugby, and handball demand rapid changes of direction, known as change of direction (COD) speed.^[1]^ Athletes are frequently required to decelerate and reaccelerate in new directions under dynamic and often unpredictable conditions. Beyond muscular strength, CoD ability reflects the integration of neuromuscular responsiveness, intermuscular coordination, and the stiffness of the muscle-tendon unit.^[2]^ Soccer players, for example, perform up to 600 directional changes per match, typically between 0° and 90°.^[3]^ Proficiency in CoD often distinguishes elite players from sub-elite counterparts, making it crucial for performance evaluation and talent identification.^[4]^

The performance relevance of CoD has led to increasing emphasis on training interventions designed to optimize it.^[2,5]^ Traditionally, resistance training (RT) approaches such as squats, power cleans, and plyometrics have been employed to develop the strength and power underpinning CoD performance. Although these methods improve linear speed and vertical power, they may insufficiently address the eccentric demands of deceleration and redirection inherent in multidirectional movements.^[2,6]^ Consequently, alternative training modalities have been explored to better target these biomechanical and neuromuscular characteristics.

Flywheel resistance training (FRT) has recently emerged as a promising alternative, characterized by its use of inertial loads to induce eccentric overload. Unlike traditional resistance training (TRT), which relies on gravity-dependent external loads, FRT utilizes rotating flywheels to generate kinetic energy during the concentric phase, which the athlete must then decelerate eccentrically. This unique mechanism allows for a greater eccentric stimulus and consistent loading across the full range of motion, especially in multiplanar and sport-specific patterns.^[7,8]^ Practically, FRT may more effectively enhance braking forces and neuromuscular adaptations essential for CoD performance.

Compared to TRT, FRT appears to offer unique benefits for enhancing multidirectional movement capabilities. Recent evidence indicates that FRT elicits greater improvements in eccentric strength and CoD-related biomechanics than traditional methods.^[9,10]^ As CoD ability relies on efficiently absorbing and redirecting forces, FRT provides a sport-specific and potentially superior alternative.^[2,6]^ However, some reviews combine different eccentric interventions, obscuring FRT’s distinct effects.^[11,12]^ FRT applies inertia-driven eccentric overload across the full range of motion, enabling neuromuscular adaptations that enhance deceleration capacity and directional control – features less emphasized in traditional, gravity-dependent RT.^12-14]^

While earlier meta-analyses have explored RT strategies for improving CoD performance,^[5,10]^ tfew have isolated FRT as a distinct modality. The only direct comparison between FRT and TRT to date was limited by a small number of studies, high heterogeneity, and a lack of moderator analysis, limiting its practical relevance.^[9]^ With several new randomized controlled trials (RCTs) now available, an updated synthesis is warranted. Therefore, this systematic review and meta-analysis aims to compare the effects of FRT and TRT on CoD performance in male team sport athletes and examine potential moderating variables such as training frequency, volume, and duration. The findings aim to bridge current knowledge gaps and inform evidence-based RT practices in multidirectional sport contexts.

## 2. Materials and methods

This systematic review was conducted according to the preferred reporting items for systematic review and meta-analysis statements.^[15]^

### 2.1. Study selection criteria

Studies were included in this systematic review if they met the following PICOS criteria – Populations: healthy individuals of any age or sex, with no recent history of musculoskeletal injury that would confound performance outcomes; Intervention: the intervention involved FRT, also described as isoinertial training; Comparison: a control group (e.g., passive control, no intervention) or an active comparison group (e.g., traditional RT) was included; Outcomes: at least 1 measure of CoD performance was reported (e.g., *T*-test, 505 agility test, pro-agility shuttle); Study Design: the study was a RCT or a randomized crossover trial.

Studies that met any of the following criteria were excluded from the review: the FRT intervention was combined with other training modalities in a way that its specific effects on CoD could not be isolated; the study was not an original peer-reviewed research article (e.g., review articles, meta-analyses, editorials, letters, conference abstracts, books, or dissertations); and the full-text was not available in English.

### 2.2. Search strategy

A keyword search was performed in 8 electronic bibliographic databases: SPORTDiscus, PubMed, Web of Science, Scopus, Medline and Google Scholar. The search algorithm included all possible combinations of keywords from the following 2 groups: “eccentric training,” “flywheel,” “FRTEO,” “flywheel resistance,” “flywheel device,” “flywheel training,” “inertial training” or “isoinertial training”; and “agility,” “change of direction” or “CoD.” Titles and abstracts of the articles identified through the keyword search were screened against the study selection criteria. Potentially relevant articles were retrieved for an evaluation of the full-text. Two coauthors independently conducted the title and abstract screening and identified potentially relevant articles for the full-text review. Interrater agreement was assessed using the Cohen’s kappa (κ = 0.92). Discrepancies were resolved through face-to-face discussions between the 2 coauthors. Articles identified from the title and abstract screening were reviewed in full texts. The 2 coauthors jointly determined the final pool of articles included in the review.

A reference list search (i.e. backward reference search) and cited reference search (i.e. forward reference search) were conducted based on the full-text articles that met the study selection criteria that were identified from the keyword search. Additionally, the reference lists of earlier published review articles on the topic were screened to search for further potentially relevant studies. Reference search was repeated on all newly identified articles until no additional relevant article was found.

### 2.3. Data extraction

A standardized data extraction form was used to collect methodological and outcome variables from each included study, including authors, publication year, study design, sample size, age, gender, sport, level, intervention frequency, intervention duration, measures, and main results.

### 2.4. Data analysis and synthesis

Meta-analysis was performed to estimate the pooled effect of FRT on CoD performance. Standardized pooled effect sizes (ES; i.e., Hedge’s g), denoted by the difference in standard deviations in the time of COD task completion between the FRT and the control group, were estimated. Study heterogeneity was assessed by the *I*^2^ index.^[16]^ The level of heterogeneity represented by the *I*^2^ index was interpreted as small (*I*^2^ ≤ 25%), moderate (25% < *I*^2^ ≤ 50%), large (50% < *I*^2^ ≤ 75%), or very large (*I*^2^ > 75%). A fixed-effect model would be estimated when a small or moderate heterogeneity was present, and a random-effect model (RE) would be estimated when a large or very large heterogeneity was present.

Publication bias was assessed by the Egger’s test.^[17]^ Statistical analyses were conducted by using Stata 17 version (StataCorp, College Station). All analyses used 2-sided tests, and a *P*-value < .05 was considered statistically significant. The SMDs were interpreted using the conventions outlined by Cohen (< 0.2 “trivial”; ≤ 0.2 ES < 0.5 “small,” ≥ 0.5 ES < 0.8 “moderate,” 0.8 “large”). Independent subgroup analyses were conducted for key training-related variables, including intervention duration, frequency, and total number of training sessions, to explore potential moderators. Positive SMD values indicated performance improvements.

To evaluate the robustness of the meta-analytic findings, sensitivity analyses were performed by excluding studies with lower methodological quality (i.e., National Institutes of Health quality score ≤ 5).^[18]^ The direction and magnitude of ESs remained stable, indicating that the overall conclusions were not disproportionately influenced by lower-quality studies. Fifteen studies were included in the meta-analysis.

### 2.5. Study quality assessment

We used the National Institutes of Health’s Quality Assessment Tool for Controlled Intervention Studies to assess the quality of each included study.^[19]^ This tool consists of 14 criteria assessing key aspects such as randomization, allocation concealment, blinding, intervention fidelity, outcome measurement, and follow-up. Each criterion was scored as 1 if the answer was “yes,” and 0 otherwise. A total quality score ranging from 0 to 14 was calculated for each study by summing the responses across all items.

The quality assessment was used to evaluate the internal validity of the included studies but was not used as a criterion for study inclusion or as a weighting factor in the meta-analysis. All studies contributed equally to the pooled estimates under the random-effects model, which inherently accounts for between-study variability. However, the quality scores informed sensitivity analyses to determine whether study quality had a meaningful impact on the results and to enhance the transparency of evidence synthesis. This study synthesized data from previously published reports and did not involve human participants or animals; therefore, institutional review board approval and informed consent were not required.

## 3. Results

### 3.1. Study characteristics

**Figure 1.**
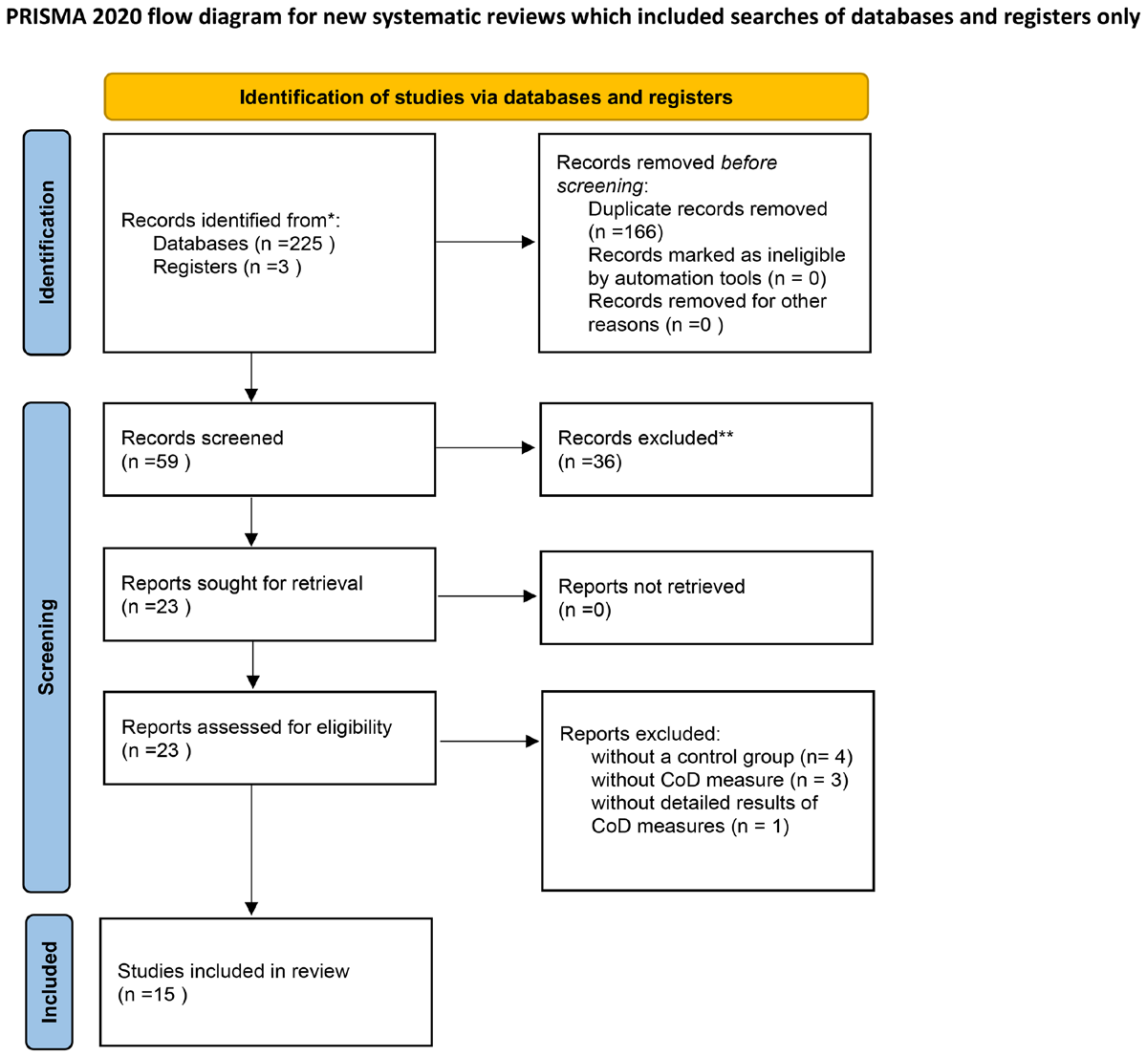
Flowchart of publications included in systematic review and meta-analysis (PRISMA diagram). COD = change-of-direction, PRISMA = Preferred Reporting Items for Systematic Reviews and Meta-Analyses.

Our literature search initially yielded 225 studies. After the removal of duplicates and the exclusion of studies based on title and abstract screening, 23 articles remained as potential candidates for inclusion. However, upon closer examination, 1 study was found to have missing data, 3 studies did not assess CoD performance, and 4 studies lacked a control group (Fig. 1). Consequently, only 15 studies met the stringent inclusion criteria, resulting in a total of 17 experimental groups. The characteristics of the reviewed articles are meticulously presented in Table 1: 14 of them were randomized controlled trials,^[7,8,11-14,20-27]^ while 1 study adopted a randomized crossover trial design.^[28]^ The total number of participants across the experimental groups ranged from 9 to 21, amounting to a cumulative total of 234 individuals. Notably, the age range of participants spanned from 13 to 24 years old. The duration of the training interventions ranged from 4 to 11 weeks, with a frequency of 1 to 3 sessions per week, culminating in a total of 8 to 24 sessions. Moreover, it is noteworthy to mention that FRT was implemented across various team sports settings, including soccer (8 studies), basketball (4 studies), and handball (3 studies), underscoring the versatility of FRT across different athletic disciplines. Additionally, the reviewed studies encompassed a balanced representation of male (10 studies) and female (5 studies) athletes, highlighting the broad applicability of FRT. Quality assessment of the studies revealed an average score of 7.5 out of 14, with scores ranging from 3 to 10, as delineated in Table 2.

**Table 1:** Basic characteristics of the studies included in the review.

Study ID	First author, yr	Study design	Sample size*	Age (yr)	Female (%)	Subjects	Intervention frequency (wk^− 1^)	Intervention duration (wk)	Total intervention sessions	Measure	Main results
1	Pecci, 2023	RCTs	EG = 12 CG = 12	EG = 20.8 ± 2.6 CG = 20.1 ± 2.6	100	Elite soccer players	2	6	12	shuttle run test	EG > CG
2	Puustinen, 2023	RCTs	EG = 9 CG = 9	EG = 18.9 ± 1.0 CG = 18.3 ± 0.5	0	Elite ice hockey players	1–2	8	10	shuttle run test	EG > CG
3	Galiano, 2022	RCTs	EG_1_ = 21 EG_2_ = 21 CG = 21	EG_1_ = 23.2 ± 2.8 EG_2_ = 22.7 ± 2.7 CG = 23.3 ± 3.7	0	Amateur soccer, volleyball, basketball and rugby players	2	6	12	V-cut test	EG1 > EG2 > CG
4	Madruga-Parera, 2022	RCTs	EG = 17 CG = 17	15.96 ± 1.39	0	Amateur handball players	3	8	24	Shuttle run test; V-cut test	EG > CG
5	Fousekis, 2021	RCTs	EG_1_ = 11 EG_2_ = 11 CG = 13	EG = 24.0 ± 6.6 CG = 23.9 ± 4.7	0	Amateur soccer player	2	6	12	IAT	EG > CG
6	Raya-González, 2021	RCTs	EG = 10 CG = 10	16	0	Elite soccer players	1	10	10	shuttle run test	EG > CG
7	Stojanovi´c, 2021	RCTs	EG = 12 CG = 12	EG = 17.6 ± 0.5 CG = 17.6 ± 0.5	0	Amateur basketball players	1–2	8	12	*T*-test	EG > CG
8	Fiorilli, 2020	RCTs	EG = 18 CG = 16	EG = 13.2 ± 1.2 CG = 13.2 ± 0.8	0	Amateur soccer players	2	6	12	Y-agility test; IAT	EG > CG
9	O Brien, 2020	RCTs	EG = 11 CG = 9	EG = 23.2 ± 5.6 CG = 24.2 ± 6.6	100	Amateur basketball players	2	4	8	shuttle run test	EG > CG
10	Coratella, 2019	RCTs	EG = 20 CG = 20	EG = 23.2 ± 5.6 CG = 24.2 ± 6. 6	0	Amateur soccer players	1	8	8	T-test; shuttle run test	EG > CG
11	Sanchez-Sanchez, 2019	RCTs	EG = 12 CG = 10	EG = 22.5 ± 2.2 CG = 22.5 ± 2.2	0	Amateur basketball and volleyball players	2	5	10	IAT	EG > CG
12	Monajati, 2018	RCTs	EG = 10 CG = 8	EG = 22.6 ± 2.3 CG = 21.0 ± 1.4	50	Amateur volleyball players	2	6	12	shuttle run test	EG > CG
13	Bourgeois, 2017	Randomized crossover trial	EG = 12 CG = 6	EG = 15.0 ± 0.9 CG = 15.3 ± 0.5	0	Amateur rugby players	3	6	18	180° and 45° COD test	EG > CG
14	Maroto-Izquierdo, 2017	RCTs	EG = 15 CG = 14	EG = 19.8 ± 1.0 CG = 23.8 ± 1.6	0	Elite handball players	2–3	6	15	*T*-test	EG > CG
15	Tous-Fajardo, 2016	RCTs	EG = 12 CG = 12	17.0 ± 0.5	0	Amateur soccer players	1	11	11	V-cut test; shuttle run test	EG > CG

CG = control group, COD = change-of-direction, EG = flywheel resistance training group, IAT = Illinois agility test, RCT = randomized controlled trial.

* Number of participants at the end of the studies.

**Table 2:** Study quality assessment.

Criteria	1	2	3	4	5	6	7	8	9	10	11	12	13	14	15
1. Was the study described as randomized, a randomized trial, a randomized clinical trial, or an RCT?	1	0	1	1	1	1	1	1	1	1	1	1	0	1	0
2. Was the method of randomization adequate (i.e., use of randomly generated assignment)?	1	0	1	1	1	1	1	1	1	1	1	0	0	1	0
3. Was the treatment allocation concealed (so that assignments could not be predicted)?	0	0	0	0	0	0	0	0	0	0	0	0	0	0	0
4. Were study participants and providers blinded to treatment group assignment?	0	0	0	0	0	0	0	0	0	0	0	0	0	0	0
5. Were the people assessing the outcomes blinded to the participants’ group assignments?	0	0	0	0	0	0	0	0	0	0	1	1	0	0	0
6. Were the groups similar at baseline on important characteristics that could affect outcomes (e.g., demographics, risk factors, co-morbid conditions)?	1	1	1	1	1	1	1	1	1	1	1	1	1	1	1
7. Was the overall drop-out rate from the study at endpoint 20% or lower of the number allocated to treatment?	1	1	1	1	1	1	1	1	1	1	1	1	0	1	1
8. Was the differential drop-out rate (between treatment groups) at endpoint 15 percentage points or lower?	1	1	1	1	1	1	1	1	1	1	1	1	0	1	1
9. Was there high adherence to the intervention protocols for each treatment group?	1	1	1	1	1	1	1	1	1	1	1	1	1	1	1
10. Were other interventions avoided or similar in the groups (e.g., similar background treatments)?	1	1	1	1	1	1	1	1	1	1	1	0	1	1	1
11. Were outcomes assessed using valid and reliable measures, implemented consistently across all study participants?	1	1	1	1	1	1	1	1	1	1	1	1	1	1	1
12. Did the authors report that the sample size was sufficiently large to be able to detect a difference in the main outcome between groups with at least 80% power?	1	0	0	0	0	1	0	0	0	0	0	1	0	0	0
13. Were outcomes reported or subgroups analyzed prespecified (i.e., identified before analyses were conducted)?	1	1	1	1	1	1	1	1	1	1	1	1	1	1	1
14. Were all randomized participants analyzed in the group to which they were originally assigned, i.e., did they use an intention-to-treat analysis?	0	1	1	1	1	0	1	1	1	1	1	0	0	1	1
Total scores	11	8	10	10	10	10	10	10	10	10	11	10	5	10	8

1 denotes Yes and 0 denotes No.

### 3.2. Meta-analysis

**Figure 2.**
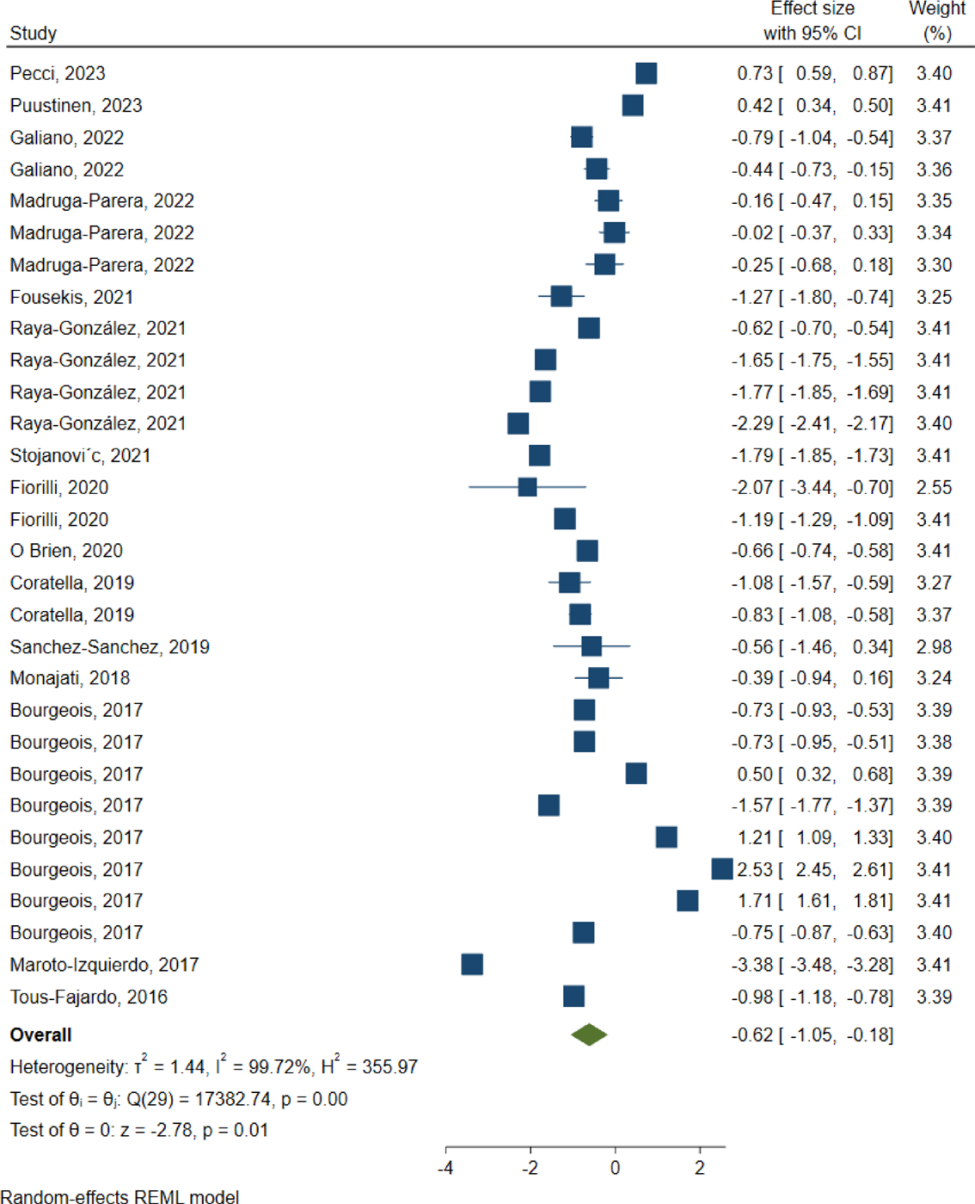
Meta-analysis of the effects of flywheel training versus traditional training. CI = confidence interval.

The results of the meta-analysis (Fig. 2) revealed a statistically significant reduction in CoD task completion time following FRT interventions, with a standardized mean difference (SMD) of −0.62 (95% confidence interval [CI] = −1.05, −0.18; *I*^2^ = 99.7%; Random Effects model). Egger’s test indicated no evidence of publication bias (*P*-value > .05). Subgroup analyses, detailed in Table 3, were conducted to explore the impact of various factors, including age, gender, sport, competitive level, training frequency and duration, and specific CoD assessment measures on the effectiveness of FRT interventions.

**Table 3:** Subgroup analysis assessing potential moderating factors for CoD performance of FRT+ vs. control group.

Population characteristics	Studies		FRT+ vs. control			
	No.	References	Pooled ES (95% CI)	*I*^2^ index (%)	*P*	*P*_diff_
Age (yr)						
≥16	13	Pecci, 2023; Puustinen, 2023; Galiano, 2022; Fousekis, 2021; Raya-González, 2021; Stojanovic, 2021; Fiorilli, 2020; O Brien, 2020; Coratella, 2019; Sanchez-Sanchez, 2019; Monajati, 2018; Maroto-Izquierdo, 2017; Tous-Fajardo, 2016	−1.07 (−1.51, −0.64)	99.60%	<.05	<.01
<16	2	Madruga-Parera, 2022; Bourgeois, 2017	0.16 (−0.56, 0.89)	99.61%	>.05	
Gender						
Female (≥50%)	3	Pecci, 2023; O Brien, 2020; Monajati, 2018	−0.10 (−0.96, 0.76)	98.91%	>.05	.25
Male	12	Puustinen, 2023; Galiano, 2022; Madruga-Parera, 2022; Fousekis, 2021; Raya-González, 2021; Stojanovi´c, 2021; Fiorilli, 2020; Coratella, 2019; Sanchez-Sanchez, 2019; Bourgeois, 2017; Maroto-Izquierdo, 2017; Tous-Fajardo, 2016	−0.67 (−1.15, −0.2)	99.73%	<.05	
Sport						
Soccer	6	Pecci, 2023; Fousekis, 2021; Raya-González, 2021; Fiorilli, 2020; Coratella, 2019; Tous-Fajardo, 2016	−1.51 (−1.64, −0.66)	99.33%	<.05	.96
Basketball	3	Stojanovi´c, 2021; O Brien, 2020; Sanchez-Sanchez, 2019	−1.06 (−1.87, −0.24)	99.46%	<.05	
Handball	2	Madruga-Parera, 2022; Maroto-Izquierdo, 2017	−0.96 (−2.56, 0.64)	99.29%	>.05	
Level						
Amateur	11	Galiano, 2022; Madruga-Parera, 2022; Fousekis, 2021; Stojanovi´c, 2021; Fiorilli, 2020; O Brien, 2020; Coratella, 2019; Sanchez-Sanchez, 2019; Monajati, 2018; Bourgeois, 2017;Tous-Fajardo, 2016	−0.42 (−0.87, 0.02)	99.51%	>.05	.18
Elite	4	Pecci, 2023; Puustinen, 2023; Raya-González, 2021; Maroto-Izquierdo, 2017	−1.22 (−2.32, −0.13)	99.90%	<.05	
Intervention characteristics						
Frequency						
≥2 wk^−1^	10	Pecci, 2023; Galiano,2022; Madruga-Parera, 2022; Fousekis, 2021; Fiorilli, 2020; O Brien, 2020; Sanchez-Sanchez, 2019; Monajati, 2018; Bourgeois, 2017; Maroto-Izquierdo, 2017	−0.37 (−0.93, 0.18)	99.64%	>.05	<.01
<2 wk^−1^	5	Puustinen, 2023; Raya-González, 2021; Stojanovi´c, 2021; Coratella, 2019; Tous-Fajardo, 2016	−1.18 (−1.71, −0.64)	99.62%	<.05	
Duration						
≤8 wk	13	Pecci, 2023; Puustinen, 2023; Galiano, 2022; Madruga-Parera, 2022; Fousekis, 2021; Stojanovi´c, 2021; Fiorilli, 2020; O Brien, 2020; Coratella, 2019; Sanchez-Sanchez, 2019; Monajati, 2018; Bourgeois, 2017; Maroto-Izquierdo, 2017	−0.44 (−0.93, 0.04)	99.68%	>.05	.01
>8 wk	2	Raya-González, 2021; Tous-Fajardo, 2016	−1.46 (−2.04, −0.08)	99.40%	<.05	
Measure						
IAT	3	Fousekis, 2021; Fiorilli, 2020; Sanchez-Sanchez, 2019	−1.19 (−1.80, −0.57)	36.49%	<.05	.03
Shuttle run test	8	Pecci, 2023; Puustinen, 2023; Madruga-Parera, 2022; Raya-González, 2021; O Brien, 2020; Coratella, 2019; Monajati, 2018; Bourgeois, 2017	−0.27 (−0.82, 0.29)	99.77%	>.05	
*T*-test	3	Stojanovi´c, 2021; Coratella, 2019; Maroto-Izquierdo, 2017	−2.10 (−3.43, −0.77)	99.76%	<.05	
V-cut test	3	Galiano, 2022; Madruga-Parera, 2022; Tous-Fajardo, 2016	−0.65 (−0.96, −0.33)	80.63%	<.05	

CI = confidence interval, CoD = change of direction, ES = effect size, FRT = flywheel resistance training, *I*^2^ = heterogeneity, IAT = Illinois agility test, No. = number of the included studies, *P* = test for overall effect, *P*_diff_ = test for subgroup differences.

The ESs of FRT interventions varied across different subgroups. Notably, adult players exhibited a more pronounced response (ES = −1.07; 95% CI = −1.51, −0.64) compared to younger counterparts under the age of 16 (ES = 0.16; 95% CI = −0.56, 0.89). Athletes undergoing less frequent training (below 2 sessions weekly) demonstrated a greater improvement (ES = −1.18; 95% CI = −1.71, −0.64) compared to those with more frequent training sessions (ES = −0.37; 95% CI = −0.93, 0.18). Longer interventions exceeding 8 weeks resulted in higher ESs (ES = −1.46; 95% CI = −2.04, −0.88) compared to shorter durations (ES = −0.44; 95% CI = −0.93, 0.04). Furthermore, FRT interventions showed greater efficacy when assessed using the *T*-test (ES = −2.10; 95% CI = -3.43, −0.77), IAT (ES = −1.19; 95% CI = −1.80, −0.57), and V-Cut Test (ES = −0.65; 95% CI = −0.96, −0.33) in comparison to the shuttle run test (ES = −0.27; 95% CI = −0.82, 0.29).

To test the robustness of the results, a sensitivity analysis was performed by excluding studies with lower methodological quality (National Institutes of Health score ≤ 5). The resulting pooled ES based on high-quality studies was − 0.95 (95% CI = −1.35, −0.55), compared to − 0.61 (95% CI = −0.64, −0.59) in the full dataset. The consistency in effect direction and increased magnitude indicate that the overall findings are robust and that the inclusion of lower-quality studies may have contributed to a conservative estimate of the true effect.

Moreover, there were no discernible differences in training effects between male and female players (*P*-value > .05), elite and amateur players (*P*-value > .05), or among soccer, basketball, and handball players (*P*-value > .05).

## 4. Discussion

This study systematically synthesized and quantified existing evidence on the effect of FRT on (CoD performance. Among the 15 studies included, comprising 14 randomized controlled trials and 1 randomized crossover trial, FRT demonstrated a clear advantage over control interventions in improving CoD performance among team sports athletes.

While these results highlight the efficacy of FRT, it is important to contextualize its performance-enhancing effects relative to other training modalities. TRT and plyometric training are also well-established methods for improving CoD through gains in power, neuromuscular control, and stretch-shortening cycle efficiency.^[7,28]^ Studies such as those by Puustinen et al^[12]^ and Fousekis et al^[22]^ reported that FRT produced comparable or moderately superior CoD gains to TRT, suggesting that FRT should be viewed as a complementary rather than universally superior method. This highlights the need for direct head-to-head comparisons in future research.

CoD performance is influenced by various factors, with eccentric strength playing a crucial role.^[2]^ FRT, by specifically enhancing eccentric strength, contributes to improved deceleration capacity and stabilization during CoD maneuvers.^[7]^ Previous studies have demonstrated a strong association between increased eccentric strength and enhanced CoD performance,^[29]^ with eccentric hamstring strength emerging as a key predictor across various sports.^[30]^ For instance, Greig and Naylor identified eccentric hamstring strength as a primary predictor of T-test performance.^[31]^ This variable may contribute to greater hip extensor torque during the penultimate step, maintaining trunk control and facilitating kinetic chain torque transfer.^[32]^ Additionally, it enhances knee stability, and optimizes braking impulse via elastic energy utilization.^[33]^

Recent studies have provided deeper insights into the neuromuscular adaptations elicited by FRT. These adaptations include enhanced motor unit recruitment, improved intermuscular coordination, increased tendon stiffness, and heightened proprioceptive feedback, as demonstrated by Galiano et al^[20]^ and Maroto-Izquierdo et al^[8]^ Collectively, these changes may contribute to reduced ground contact time and more efficient reacceleration during COD tasks. However, direct biomechanical assessments – such as electromyography (EMG), joint torque analysis, and kinematic evaluations – remain limited in the current literature and should be prioritized in future research.

Kinematic modifications, such as contact time and braking impulse, further elucidate the impact of FRT on CoD performance. Studies indicate that FRT enables athletes to achieve better deceleration performance, resulting in increased force output and improved propulsive ability during direction changes.^[34,35]^ Additionally, athletes generating greater braking forces exhibit earlier acceleration into the new direction, leading to faster CoD task completion.^[36]^

Regarding the subgroup analysis by age, adult athletes exhibited superior training adaptations following FRT compared to their adolescent counterparts. This disparity can be primarily attributed to greater musculoskeletal maturity in adults, who typically possess fully developed muscle architecture capable of handling the demands of eccentric overload.^[21,37]^ In contrast, adolescents are still undergoing muscular development, and their immature muscle fibers may limit their responsiveness to FRT stimuli. Another contributing factor is RT experience. Adults generally have more years of exposure to structured strength training, which translates into greater technical proficiency, motor control, and proprioceptive awareness – elements that are critical for executing FRT movements effectively.^[38]^ Beyond these differences, adults also tend to exhibit higher neuromuscular efficiency, defined as the ability of the nervous system to effectively recruit and synchronize motor units. This is particularly important for managing the simultaneous concentric–eccentric demands inherent in FRT.^[37,39]^ Finally, hormonal differences may contribute to the observed training disparities. Elevated concentrations of anabolic hormones – especially testosterone in male adults – facilitate greater postexercise protein synthesis, which may enhance muscular adaptation and recovery in response to FRT compared to adolescents.

Concerning training frequency, FRT interventions appear more effective when performed less than twice per week, compared to higher-frequency protocols. This finding aligns with the principle of super-compensation, wherein sufficient recovery between sessions enables optimal muscular adaptation. Due to the high eccentric loading characteristic of FRT, training frequently can induce significant muscle damage, necessitating extended recovery periods to allow for repair and remodeling.^[40]^ Low-frequency training allows athletes to recover more fully, potentially enhancing muscle adaptation and performance gains. Moreover, frequent high-intensity sessions may increase neuromuscular fatigue, which can compromise the quality of subsequent workouts and hinder overall progress. Excessive frequency – particularly when combined with regular team practices – may also elevate the risk of overtraining, thereby blunting or even reversing performance improvements.^[41,42]^ Seitz et al^[43]^ similarly suggest that elevated training frequency can impair performance outcomes due to accumulated physiological stress.

Duration is another pivotal consideration in FRT program design. Interventions exceeding 8 weeks manifest superior outcomes compared to those lasting 8 weeks or less. Brief interventions might inadequately catalyze neuromuscular and mechanical adaptations for positive CoD performance enhancement. Longer training durations enable more substantive muscular transformations. Tesch et al^[44]^ advocate that RT induces hypertrophy and strength gains, suggesting that prolonged FRT might intensify these benefits. Extended interventions can also heighten neuromuscular efficiency.^[45]^ And while FRT’s eccentric component can cause significant muscle damage, enduring interventions can instigate multiple damage-repair cycles, promoting a potentiated super-compensation mechanism.^[46]^ Longer training durations with FRT may also allow athletes to master the technique and harness the full benefits of the modality, which is crucial for optimizing outcomes.^[47]^

Regarding performance metrics, FRT interventions yielded more substantial improvements in the T-test, Illinois agility test (IAT), and V-cut test compared to the shuttle run test. This disparity likely reflects the distinct neuromuscular and mechanical demands of these assessments, as well as the specific adaptations promoted by FRT. The Shuttle Run Test, which involves repeated 180° turns, places considerable emphasis on dynamic balance, turning proficiency, and inter-limb coordination. These elements require not only muscular strength but also fine motor control, rapid reorientation, and spatial awareness. Although FRT effectively enhances muscular power and eccentric strength, its benefits may not directly transfer to the complex motor patterns and aerobic components emphasized in the shuttle run.^[48]^ In contrast, the T-test, IAT, and V-cut test primarily involve shorter, explosive direction changes that align more closely with the anaerobic and eccentric overload characteristics of FRT. These tests are less reliant on prolonged aerobic capacity and more sensitive to the neuromuscular gains induced by FRT. Therefore, while FRT is effective for improving power and directional control, integrating complementary training focused on balance, agility, and skill-specific drills may be necessary to optimize performance across a broader range of CoD assessments.

### 4.1. Limitations

Despite encouraging findings, this review has several limitations. Most included studies had small sample sizes and short durations, limiting statistical power and insights into long-term effects. Substantial heterogeneity – evidenced by elevated *I*^2^ values – was also present, likely due to variability in participant characteristics, FRT protocols, and CoD assessments, which constrains comparability and generalizability. Specifically, differences in flywheel device models, resistance settings, training execution techniques, and athlete profiles may have contributed significantly to the observed heterogeneity. Moreover, current evidence is primarily drawn from soccer, basketball, and handball athletes, limiting applicability to contact-heavy sports such as rugby or American football, which entail distinct neuromechanical demands. While Sabido et al^[49]^ offer initial data in rugby, broader sport-specific validation is needed.

### 4.2. Future perspectives

Future research should adopt larger, more diverse samples, standardized interventions, and consistent outcome measures. Longitudinal studies are essential to assess sustained effects, and the inclusion of perceptual and reactive agility assessments would enhance ecological validity and better reflect real-world sport performance. In addition, trials should be prospectively registered and adequately powered, use volume/intensity-matched comparators, and standardize FRT reporting (device/model, moment of inertia, set–rep–rest, progression, familiarization, adherence). A core COD test set distinguishing preplanned vs reactive tasks and specifying turn angles (e.g., 45°, 90°, 180°), plus deceleration metrics (approach speed, braking impulse, contact time) via timing gates/IMUs/force plates, is recommended. Priority samples include women, youth, and contact-heavy sports; studies should report adverse events and examine transfer to competition and long-term retention.

## 5. Conclusion

FRT emerges as an effective intervention for enhancing CoD performance in team sports athletes. Lower training frequencies (<2 sessions/wk) combined with extended durations (exceeding 8 weeks) prove to be the most efficacious approach. These findings provide valuable insights for coaches and strength and conditioning professionals in designing optimal training programs for team sports athletes.

## Acknowledgments

We gratefully acknowledge institutional support from the Key Laboratory of Sport Training of the General Administration of Sport of China and the International Joint Laboratory on High-Performance Sports Research.

## Author contributions

**Data curation:** Junxin Zhang.

**Formal analysis:** Junxin Zhang.

**Methodology:** Junxin Zhang, Ruidong Liu.

**Writing – original draft:** Junxin Zhang.

**Writing – review & editing:** Jing Mi, Ruidong Liu.

## References

[1] Loturco I Pereira LA Reis VP. Change of direction performance in elite players from different team sports. J Strength Cond Res. 2022;36:862–6. 32168177 10.1519/JSC.0000000000003502

[2] Brughelli M Cronin J Levin G Chaouachi A. Understanding change of direction ability in sport: a review of resistance training studies. Sports Med (Auckland, N.Z.)Sports Med. 2008;38:1045–63. 10.2165/00007256-200838120-00007 19026020

[3] Bloomfield J Polman R O’Donoghue P. Physical demands of different positions in FA premier league soccer. J Sports Sci Med. 2007;6:63–70. 24149226 PMC3778701

[4] Unnithan V White J Georgiou A Iga J Drust B. Talent identification in youth soccer. J Sports Sci. 2012;30:1719–26. 23046427 10.1080/02640414.2012.731515

[5] Asadi A Arazi H Young WB Sáez de Villarreal E. The effects of plyometric training on change-of-direction ability: a meta-analysis. Int J Sports Physiol Perform. 2016;11:563–73. 27139591 10.1123/ijspp.2015-0694

[6] Lockie RG Schultz AB Callaghan SJ Jeffriess MD. The effects of traditional and enforced stopping speed and agility training on multidirectional speed and athletic function. J Strength Cond Res. 2014;28:1538–51. 24169474 10.1519/JSC.0000000000000309

[7] Tous-Fajardo J Gonzalo-Skok O Arjol-Serrano JL Tesch P. Enhancing change-of-direction speed in soccer players by functional inertial eccentric overload and vibration training. Int J Sports Physiol Perform. 2016;11:66–73. 25942419 10.1123/ijspp.2015-0010

[8] Maroto-Izquierdo S García-López D de Paz JA. Functional and muscle-size effects of flywheel resistance training with eccentric-overloadin professional handball players. J Hum Kinet. 2017;60:133–43. 29339993 10.1515/hukin-2017-0096 PMC5765793

[9] Chaabene H Markov A Prieske O. Effect of flywheel versus traditional resistance training on change of direction performance in male athletes: a systematic review with meta-analysis. Int J Sports Physiol Perform. 2022;19:7061. 10.3390/ijerph19127061 PMC9223129 35742311

[10] Liu R Liu J Clarke CV An R. Effect of eccentric overload training on change of direction speed performance: a systematic review and meta-analysis. J Sports Sci. 2020;38:2579–87. 32677542 10.1080/02640414.2020.1794247

[11] Brien JO Browne D Earls D. The effects of different types of eccentric overload training on strength, speed, power and change of direction in female basketball players. J Funct Morphol Kinesiol. 2020;5:50. 33467266 10.3390/jfmk5030050 PMC7739370

[12] Puustinen J Venojärvi M Haverinen M Lundberg TR. Effects of flywheel vs. traditional resistance training on neuromuscular performance of elite ice hockey players. J Strength Cond Res. 2023;37:136–40. 36515598 10.1519/JSC.0000000000004159

[13] Pecci J Muñoz-López A Jones P Sañudo B. Effects of 6 weeks in-season flywheel squat resistance training on strength, vertical jump, change of direction and sprint performance in professional female soccer players. Biol Sport. 2023;40:521–9. 37077802 10.5114/biolsport.2023.118022 PMC10108773

[14] Fiorilli G Mariano I Iuliano E. Isoinertial eccentric-overload training in young soccer players: effects on strength, sprint, change of direction, agility and soccer shooting precision. J Sports Sci Med. 2020;19:213–23. 32132845 PMC7039027

[15] Moher D Liberati A Tetzlaff J Altman DG; PRISMA Group. Preferred reporting items for systematic reviews and meta-analyses: the PRISMA statement. Ann Intern Med. 2009;151:264–9, W64. 19622511 10.7326/0003-4819-151-4-200908180-00135

[16] Huedo-Medina TB Sánchez-Meca J Marín-Martínez F Botella J. Assessing heterogeneity in meta-analysis: Q statistic or I2 index. Psychol Methods. 2006;11:193–206. 16784338 10.1037/1082-989X.11.2.193

[17] Egger M Smith GD Schneider M. Bias in meta-analysis detected by a simple, graphical test. BMJ. 1997;315:629–34. 9310563 10.1136/bmj.315.7109.629 PMC2127453

[18] Page MJ McKenzie JE Bossuyt PM. The PRISMA 2020 statement: an updated guideline for reporting systematic reviews. BMJ (Clinical research ed.). 2021;372:n71. 10.1136/bmj.n71 PMC8005924 33782057

[19] NIH, National Heart, Lung, and Blood Institute. Quality assessment tool for before-after (pre-post) studies with no control group. 2014.

[20] Galiano C Floria P Muñoz-López A Sáez de Villarreal E Nuñez FJ. Stable vs. variable eccentric load. Do they induce different training and physical performance outcomes? Eur J Sport Sci. 2023;23:1932–9. 36017685 10.1080/17461391.2022.2118081

[21] Madruga-Parera M Bishop C Fort-Vanmeerhaeghe A Beato M Gonzalo-Skok O Romero-Rodríguez D. Effects of 8 Weeks of Isoinertial vs. Cable-Resistance Training on Motor Skills Performance and Interlimb Asymmetries. J Strength Cond Res. 2022;36:1200–8. 32379241 10.1519/JSC.0000000000003594

[22] Fousekis A Fousekis K Fousekis G. The effects of free weights and isoinertial resistance during semisquatting exercise on amateur soccer players’ physical performance indicators: a randomized controlled study. J Sports Med Phys Fitness. 2022;62:609–17. 33871246 10.23736/S0022-4707.21.12281-9

[23] Raya-González J Castillo D de Keijzer KL Beato M. The effect of a weekly flywheel resistance training session on elite U-16 soccer players’ physical performance during the competitive season. A randomized controlled trial. Res Sports Med. 2021;29:571–85. 33401975 10.1080/15438627.2020.1870978

[24] Stojanović MDM Mikić M Drid P. Greater power but not strength gains using flywheel versus equivolumed traditional strength training in junior basketball players. Int J Environ Res Public Health. 2021;18:1181. 33572738 10.3390/ijerph18031181 PMC7908554

[25] Coratella G Beato M Cè E. Effects of in-season enhanced negative work-based vs traditional weight training on change of direction and hamstrings-to-quadriceps ratio in soccer players. Biol Sport. 2019;36:241–8. 31624418 10.5114/biolsport.2019.87045 PMC6786325

[26] Sánchez-Sánchez J Gonzalo-Skok O Carretero M Pineda A Ramirez-Campillo R Yuzo Nakamura F. Effects of concurrent eccentric overload and high-intensity interval training on team sports players’ performance. Kinesiology (Zagreb, Online). 2019;51:119–26.

[27] Monajati A Larumbe-Zabala E Goss-Sampson M Naclerio F. Injury prevention programs based on flywheel vs. body weight resistance in recreational athletes. J Strength Cond Res. 2021;35:S188–96. 30273287 10.1519/JSC.0000000000002878

[28] Bourgeois FA Gamble P Gill ND McGuigan MR. Effects of a six-week strength training programme on change of direction performance in youth team sport athletes. Sports (Basel). 2017;5:83. 29910443 10.3390/sports5040083 PMC5969044

[29] Brughelli M Cronin J Levin G Chaouachi A. Understanding change of direction ability in sport. Sports Med. 2008;38:1045–63. 19026020 10.2165/00007256-200838120-00007

[30] Spiteri T Nimphius S Hart NH Specos C Sheppard JM Newton RU. Contribution of strength characteristics to change of direction and agility performance in female basketball athletes. J Strength Cond Res. 2014;28:2415–23. 24875426 10.1519/JSC.0000000000000547

[31] Greig M Naylor J. The efficacy of angle-matched isokinetic knee flexor and extensor strength parameters in predicting agility test performance. Int J Sports Phys Ther. 2017;12:728–36. 29181250 PMC5685408

[32] Jones P Thomas C Dos’Santos T McMahon J Graham-Smith P. The role of eccentric strength in 180° turns in female soccer players. Sports. 2017;5:42. 29910402 10.3390/sports5020042 PMC5968983

[33] Norrbrand L Pozzo M Tesch PA. Flywheel resistance training calls for greater eccentric muscle activation than weight training. Eur J Appl Physiol. 2010;110:997–1005. 20676897 10.1007/s00421-010-1575-7

[34] de Hoyo M Sañudo B Carrasco L. Effects of 10-week eccentric overload training on kinetic parameters during change of direction in football players. J Sports Sci. 2016;34:1380–7. 26963941 10.1080/02640414.2016.1157624

[35] Spiteri T Cochrane JL Hart NH Haff GG Nimphius S. Effect of strength on plant foot kinetics and kinematics during a change of direction task. Eur J Sport Sci. 2013;13:646–52. 24251742 10.1080/17461391.2013.774053

[36] Green BS Blake C Caulfield BM. A comparison of cutting technique performance in rugby union players. J Strength Cond Res. 2011;25:2668–80. 21886011 10.1519/JSC.0b013e318207ed2a

[37] Folland JP Williams AG. The adaptations to strength training : morphological and neurological contributions to increased strength. Sports Med. 2007;37:145–68. 17241104 10.2165/00007256-200737020-00004

[38] Maszczyk A Wilk M Krzysztofik M. The effects of resistance training experience on movement characteristics in the bench press exercise. Biol Sport. 2020;37:79–83. 32205913 10.5114/biolsport.2019.83008 PMC7075231

[39] Deschenes MR Kraemer WJ. Performance and physiologic adaptations to resistance training. Am J Phys Med Rehabil. 2002;81:S3–16. 12409807 10.1097/00002060-200211001-00003

[40] Fleck SJ. Periodized strength training: a critical review. J Strength Cond Res. 1999;13:82–9.

[41] Kreher JB Schwartz JB. Overtraining syndrome: a practical guide. Sports Health. 2012;4:128–38. 23016079 10.1177/1941738111434406 PMC3435910

[42] Proske U Morgan DL. Muscle damage from eccentric exercise: mechanism, mechanical signs, adaptation and clinical applications. J Physiol. 2001;537:333–45. 11731568 10.1111/j.1469-7793.2001.00333.x PMC2278966

[43] Seitz LB Reyes A Tran TT Saez de Villarreal E Haff GG. Increases in lower-body strength transfer positively to sprint performance: a systematic review with meta-analysis. Sports Med. 2014;44:1693–702. 25059334 10.1007/s40279-014-0227-1

[44] Tesch PA Fernandez-Gonzalo R Lundberg TR. Clinical applications of iso-inertial, eccentric-overload (YoYo™) resistance exercise. Front Physiol. 2017;8:241. 28496410 10.3389/fphys.2017.00241 PMC5406462

[45] Aagaard P Simonsen EB Andersen JL Magnusson P Dyhre-Poulsen P. Increased rate of force development and neural drive of human skeletal muscle following resistance training. J Appl Physiol (1985). 2002;93:1318–26. 12235031 10.1152/japplphysiol.00283.2002

[46] Schoenfeld BJ. Does exercise-induced muscle damage play a role in skeletal muscle hypertrophy? J Strength Cond Res. 2012;26:1441–53. 22344059 10.1519/JSC.0b013e31824f207e

[47] Dankel SJ Mattocks KT Jessee MB. Frequency: the overlooked resistance training variable for inducing muscle hypertrophy? Sports Med. 2017;47:799–805. 27752983 10.1007/s40279-016-0640-8

[48] Castagna C Abt G D’Ottavio S. Physiological aspects of soccer refereeing performance and training. Sports Med. 2007;37:625–46. 17595157 10.2165/00007256-200737070-00006

[49] Sabido R Pombero L Hernández-Davó JL. Differential effects of low vs. high inertial loads during an eccentric-overload training intervention in rugby union players: a preliminary study. J Sports Med Phys Fitness. 2019;59:1805–11. 30990262 10.23736/S0022-4707.19.09425-8

